# Therapeutic Doll Interventions for People Living with Dementia in Care Homes: A Scoping Review

**DOI:** 10.3390/nursrep14040200

**Published:** 2024-10-01

**Authors:** Elizabeth Henderson, Hannah McConnell, Gary Mitchell

**Affiliations:** School of Nursing and Midwifery, Queen’s University Belfast, Belfast BT9 7BL, UK; h.mcconnell@qub.ac.uk (H.M.); gary.mitchell@qub.ac.uk (G.M.)

**Keywords:** doll therapy, dementia, nursing, care homes, non-pharmacological interventions, BPSD, agitation, interventions, health, scoping review

## Abstract

Doll therapy is a psychosocial intervention that has been commonly used within dementia care for many decades. The practice of doll therapy involves supporting people with dementia to engage with a therapeutic doll and this has been associated with positive changes in a person’s wellbeing and behavior. While there have been several systematic reviews that have focused on behavioral outcomes, limited reviews have explored the broader psychosocial outcomes associated with doll therapy in care home settings. A scoping review of the literature was conducted following the Preferred Reporting Items for Systematic Reviews and Meta-Analysis Extension for Scoping Reviews (PRISMA-ScR). Four electronic databases were searched systematically (CINAHL Plus, Medline, PsycINFO, and PubMed). Twelve primary research studies from the past ten years (2013–2023) were included in this review. Primary data were synthesized using a narrative synthesis methodology. The three main themes from this review relate to a reduction in levels of behavioral and psychological symptoms of dementia (BPSD), increased communication skills, and a reduction in caregiver distress. Doll therapy has the potential to be an effective person-centered intervention that can enhance quality of life for people living with dementia in care home settings.

## 1. Introduction

Globally, there are currently more than 55 million people living with dementia, over 60% of whom live in low- and middle-income countries. Every year, there are nearly 10 million new cases [1]. The severity of the challenge, coupled with the imperative to proactively address prevention, diagnosis, support, and the overall quality of life throughout the dementia journey, is expected to increase significantly [2].

Given the limited effectiveness of existing pharmacological interventions and recognition that care should be individualized and person-centered, research has increasingly turned its focus to non-pharmacological interventions [3]. Often, these approaches prove sufficient in reducing episodes of distress and promoting sustained levels of wellbeing [4]. They hold the potential to enhance functionality, independence, and quality of life. Additionally, non-pharmacological interventions are non-invasive, safe, and tend to have minimal side effects [5].

In care homes, where many individuals with dementia reside, implementing non-pharmacological interventions like doll therapy may significantly impact the quality of care and life for residents. Care homes provide a controlled environment where staff can consistently apply such therapies, monitor outcomes, and adjust care plans as needed [6]. The structured setting of care homes also facilitates the incorporation of doll therapy into daily routines, thereby promoting a sense of normalcy and routine for residents [7]. Doll therapy in care homes can contribute to reducing the reliance on medication for managing behavioral and psychological symptoms of dementia, contributing to a more holistic and person-centered approach to care [8].

Doll therapy is a type of non-pharmacological approach for maintaining wellbeing or alleviating distress in certain individuals living with dementia [6]. This therapy involves individuals with dementia engaging with a doll through various forms of interaction, such as holding, talking to, cuddling, hugging, feeding, or dressing the doll [7]. The incorporation of dolls as a therapeutic intervention for dementia patients is linked to the concept of attachment [6]. This association draws from attachment theory, initially developed by John Bowlby to comprehend the challenges faced by children separating from their parents [8]. In the context of dementia, dolls can take on the symbolic importance of a real baby, allowing for individuals to redirect their caregiving and protection instincts towards nurturing behaviors [9]. Doll therapy establishes a therapeutic bond, fosters a sense of calmness and competence, offers sensory stimulation, and plays a role in enhancing communication with both the patient’s external environment and caregivers [7].

Doll therapy has shown promising results in dementia care [10,11,12,13]. Studies have demonstrated that doll therapy can effectively reduce the behavioral and psychological symptoms of dementia (BPSD), improve communication [10] and social interactions [13], and decrease distressing behaviors such as wandering [12]. Additionally, doll therapy has been associated with positive emotional responses, including laughter and improved verbal communication [13]. The benefits of doll therapy extend to both male and female individuals with dementia, highlighting its potential for diverse populations. Overall, the evidence suggests that doll therapy is a valuable non-pharmacological intervention that can enhance the quality of life for individuals living with dementia by promoting feelings of security, engagement, and improved social interactions.

Given these promising results, it is essential to further explore the broader implications and potential of doll therapy in dementia care. The rationale for this scoping review on doll therapy in dementia, despite existing systematic reviews [6,8,12], lies in the need to address an unexplored area in the literature. While previous systematic reviews have examined specific outcomes of doll therapy, they have not focused specifically on its impact within care home settings. Nurses play a pivotal role in care home settings, often serving as the primary healthcare providers and the main link to other multidisciplinary team services that are typically offsite. Unlike hospitals, care homes rarely have doctors on-site, which places nurses at the forefront of implementing and managing interventions for residents with dementia. This unique position makes it crucial for nurses to be empowered with evidence-based, non-pharmacological interventions like doll therapy. By conducting this review, the authors aim to provide nurses with evidence on the effectiveness of doll therapy in care home settings, enabling them to make informed decisions and lead person-centered care initiatives for residents with dementia. The review question is, what is the impact of doll therapy interventions on people living with dementia in care home settings?

Therefore, this scoping review aims to examine the evidence specifically within care home settings, identifying gaps that may have been overlooked in previous systematic reviews, which have considered all types of settings. By focusing on care homes, the authors aim to present findings that are unique to these settings and that differ from those reported by authors in broader reviews.

## 2. Materials and Methods

### 2.1. Study Design

This scoping review (ScR) employed a systematic approach, adhering to guidelines from the Joanna Briggs Institute (JBI) [14], Arksey and O’Malley [15], and the Preferred Reporting Items for Systematic Reviews and Meta-Analysis Extension for ScR (PRISMA-ScR) [16]. The process included formulating a research question, identifying relevant studies by developing eligibility criteria, and selecting pertinent studies. Subsequently, data were analyzed and extracted, with the results summarized and reported. Please see Supplementary Material Table S1 for a copy of the PRISMA-ScR checklist.

### 2.2. Search Method

Four electronic databases were chosen and searched in June 2024: CINAHL, Medline, PsycINFO, and PubMed. The selection of databases for this review was based on their comprehensive coverage of the medical, psychological, and nursing literature. These databases collectively provide a well-rounded perspective on the multidimensional aspects of doll therapy in dementia. Key search terms were developed following an initial review of the literature and were subsequently adapted to align to the primary aim of this integrative review. Search terms were informed by previous systematic reviews on doll therapy [6,8,12]. Terms were also reviewed by a subject librarian and two people with experience in reviews and this topic.

The database search involved the following key search terms as MESH terms or free-text terms and were linked together with the AND and OR Boolean operators: (a) Dementia OR Alzheimer’s disease OR People with dementia OR Living with Dementia OR Cognitive Impairment and (b) Doll Therapy OR Doll OR Dolls OR Soft Toy OR Baby Doll OR Empathy Doll. EndNote, version 21 (Clarivate, Philadelphia, USA) was used to combine the searches from the four electronic databases and was used to remove the duplicates. Please see Supplementary Material Table S2 for an example of the search results from CINAHL.

### 2.3. Inclusion and Exclusion Criteria

The population, exposure, and outcome (PEO) framework was first used to develop inclusion criteria as recommended by Bettany-Salttikov and McSherry [17]. This review included all types of empirical studies and evidence reviews (e.g., systematic reviews, quantitative research, qualitative research, mixed methods research). Typically, a PEO research question is employed when both the population and exposure are clearly defined, with measurable outcomes. However, given the limited available literature and in alignment with prior literature reviews in the field [8], a PE format was adopted.

While this scoping review only includes studies written in English, no geographical restrictions were applied. Studies were limited to those published in the last ten years due to previous systematic reviews on the topic. Studies focusing on individuals with dementia who received doll therapy in care homes or long-term care facilities were included. Studies that included acute care or that were hospital-based were excluded. In cases where doll therapy was examined alongside another intervention, data were extracted only for the doll therapy intervention.

### 2.4. Data Extraction

First-level screening included only the title and abstract only by EH and GM. Preliminary exclusions ruled out papers that did not relate to doll therapy or reported on child populations and play therapy. Non-English language papers were also screened out at this first stage. Full-text articles were then reviewed to assess eligibility. Exclusion criteria at this second stage included commentaries and anecdotal papers on doll therapy, pre-2014 papers, non-peer-reviewed papers or dissertations, case reports, and non-empirical research. Twelve papers remained at this part of the selection process. The last screening was hand-searching all full text papers cross-referencing the papers on Google Scholar. Based on the inclusion and exclusion criteria, there were no further papers that met the criteria. All stage 1 and stage 2 screenings were undertaken independently (EH and GM). A third independent reviewer was involved when there was a query involving inclusion and exclusion of a paper (HM).

### 2.5. Narrative Synthesis

Data were analyzed using a narrative synthesis approach. This method involves examining data relationships by organizing and categorizing findings from the included studies to identify and describe recurring patterns [18]. Narrative synthesis is particularly useful when studies in a review are diverse in terms of research design, methodologies, and outcomes [19]. It allows for the integration of both qualitative and quantitative data, providing a flexible approach for summarizing and interpreting research findings [20]. To ensure the robustness of the synthesis process, EH maintained a detailed diary to document and reflect upon the synthesis process. This diary served a dual purpose: encouraging self-reflection to consider personal biases and enhancing transparency by recording the steps taken during the review.

The data synthesis occurred in three phases: In phase 1, data extraction involved systematically summarizing each selected study using a standardized template. This template ensured consistency and comprehensiveness in capturing essential study details. During phase 2, thematic analysis of the extracted data were conducted. This phase involved identifying and coding significant themes across the studies, allowing for the aggregation of similar findings. Finally, in phase 3, the descriptive themes identified in phase 2 were refined and synthesized into three overarching themes. These themes were developed and refined to provide a narrative of the findings [18,19,20].

## 3. Results

**Figure 1:**
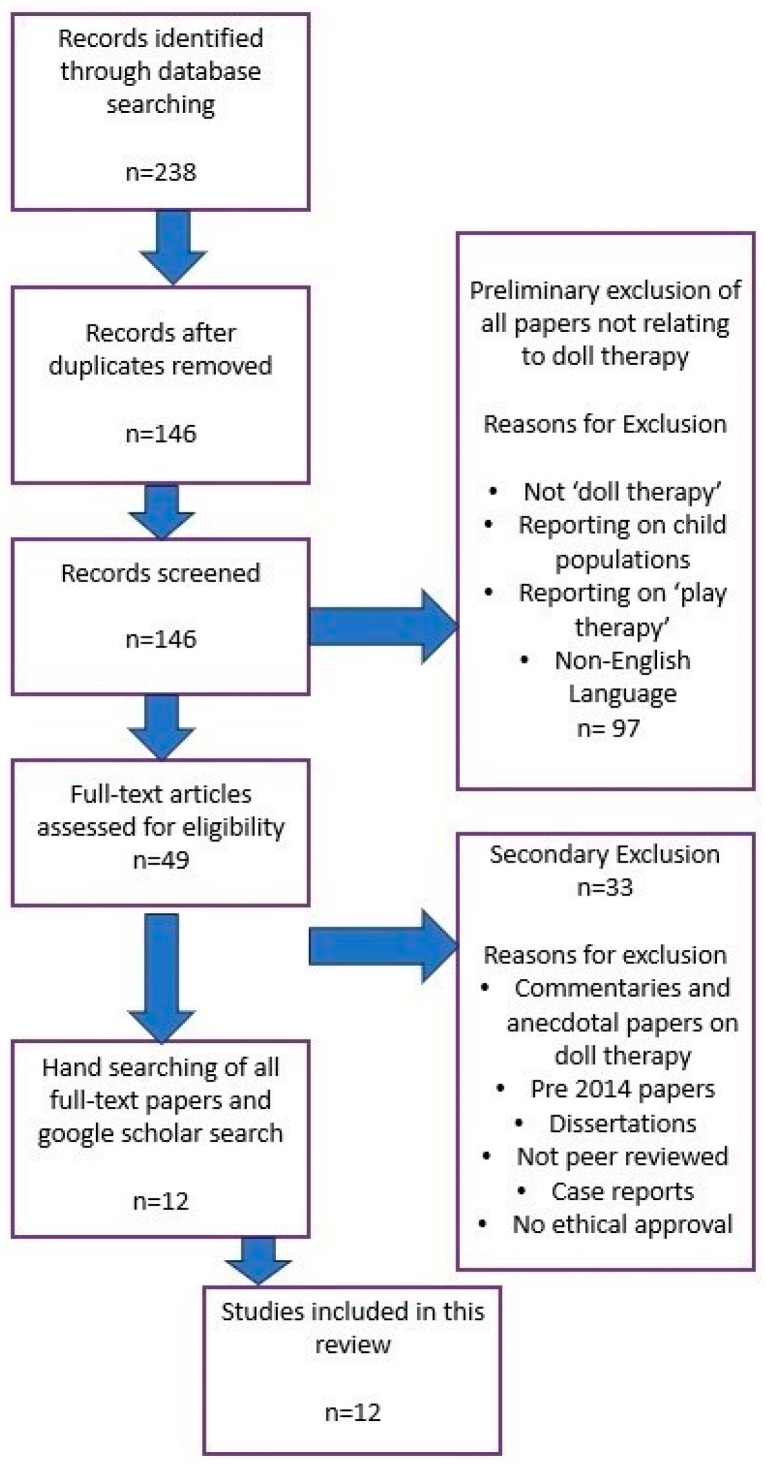
PRISMA flow diagram. PRISMA: Preferred Reporting Items for Scoping Reviews and Meta-Analyses.

The search yielded 145 papers following the removal of duplicates. Following screening of title and abstracts, 49 full-text papers were reviewed. Following full-text review, 12 studies met the inclusion criteria and were included in this review [21,22,23,24,25,26,27,28,29,30,31,32]. The PRISMA flow chart summarizes the above process below in Figure 1.

### 3.1. Characteristics of Included Studies

Twelve papers met the inclusion criteria. Eight were quantitative, including six randomized controlled trials (RCTs) [22,24,27,29,31,32]. These RCTs used doll therapy as the intervention. Five of these studies used one or more control groups, including standard clinical treatment [29], a soft toy-like cube [27,32], hand warmers with sensory characteristics [24], and a non-anthropomorphic object [31]. One study included three groups: control, doll therapy, and gesture verbal treatment [22]. In addition to these, two experimental designs were included [23,30]. One study adopted a pre/post-test design measuring behavior, mood, and social interaction [30], while the other assessed happiness, agitation, and ease in caregiving as primary outcomes [23].

The remaining four studies included one mixed-methods study [28] and three qualitative studies [21,25,26]. All these studies employed some form of semi-structured interview with one study using non-participant observation [26]. Table 1 provides an overview of the characteristics of the included studies.

### 3.2. Synthesis of Evidence

The included studies explored and examined the holistic needs of individuals living with dementia and the effectiveness of doll therapy in care home settings. From a narrative synthesis of the data, three main themes emerged: a reduction in levels of behavioral and psychological symptoms of dementia (BPSD), increased communication skills, and a reduction in caregiver distress.

#### 3.2.1. Theme 1: Doll Therapy and Behavioral and Psychological Symptoms of Dementia

The most consistent observation is the notable reduction in behavioral and psychological symptoms of dementia (BPSD) among individuals engaging in doll therapy within care homes. Eleven authors in this review explicitly report significant decreases in these negative behaviors, including reductions in episodes of verbal and physical aggression, agitation, and wandering [22,23,24,25,26,27,28,29,30,31,32]. Furthermore, another author suggested there was a tranquilizing effect experienced by participants in their qualitative study, although specific behavioral manifestations were not extensively explored [21].

Six studies in this review utilized the validated Neuropsychiatric Inventory (NPI) to evaluate the impact of doll therapy on behavioral symptoms in care homes [22,24,27,29,31,32]. The NPI assesses twelve distinct areas, with review findings consistently indicating positive trends toward reducing agitation and irritability following doll therapy. Two studies presented results across these twelve areas, revealing statistically significant reductions in agitation, apathy, and depression [22,29]. This was reflected in reductions in the mean NPI score post-intervention in two further studies: 58.7 to 34.7 [32] and 33.84 to 21.1 [27]. Overall, these studies utilized a validated instrument to illustrate statistically significant reductions in behavioral and psychological symptoms of dementia for residents in care homes using doll therapy in comparison to their control group.

However, it is important to acknowledge that not all responses to doll therapy were uniformly positive. One qualitative study reported instances where a participant displayed negative reactions to doll therapy, particularly when the doll was placed in precarious positions, causing distress or confusion as the participant rushed to cradle the doll as if it were a real baby [26]. This highlights a potential concern regarding the misinterpretation of the doll’s role or actions, which may lead to distress and increase behavioral and psychological symptoms of dementia.

Overall, the literature suggests psychological changes accompanying the positive behavioral effects of doll therapy. A qualitative study in a European long-term care facility discussed how doll therapy can enhance purpose, responsibility, and pride among participants, fostering a sense of fulfillment and agency [21]. Another study in a long-term care facility in the Far East noted significant improvements in positive mood and decreases in depression levels following doll therapy [30]. The identification of happiness or well-being as a key psychological benefit and reduction of apathy were also key findings from quantitative studies included in this review [22,23,29].

While most studies were atheoretical, two studies [21,31] specifically addressed attachment theory, a cornerstone in the doll therapy literature, to explain the mechanisms underlying its therapeutic benefits within the care home. Attachment theory, originally developed by John Bowlby, suggests that all people have an inherent need to form close emotional bonds. In the context of dementia care, the studies suggest doll therapy supports this need in people living with dementia in care home settings because it could reduce anxiety, promote feelings of security, and improve emotional wellbeing [21,31]. These studies therefore highlight how the emotional connections formed with the dolls in care homes could mitigate feelings of loneliness and isolation, fostering a sense of attachment and safety among individuals with dementia [21,31].

Some studies in this review also demonstrated changes in behavioral and psychological symptoms through physiological measures [27,31,32]. One study demonstrated a significant improvement in Mini-Mental State Examination (MMSE) scores within their doll intervention group, suggesting a limited positive impact on cognitive functioning [32]. Two other studies examined the impact of doll therapy by measuring biomarkers such as blood pressure, heart rate, and cortisol levels before and after interventions [27,31]. Although these studies provided some evidence of promise regarding the physiological effects of doll therapy, they did not find statistically significant results. This indicates that while doll therapy may offer cognitive and psychological benefits, its physiological effects require further investigation to establish conclusive evidence.

#### 3.2.2. Theme 2: Doll Therapy and Communication

Communication emerged as a pivotal theme across all twelve studies, highlighting the potential of doll therapy on various facets of communication among individuals with dementia in care homes. This theme includes the initiation of communication, improvements in communication quality, and enhanced interactions with staff and caregivers.

One qualitative study from the United Kingdom provided a comprehensive portrayal of how doll therapy facilitates communication among residents with dementia [21]. The study showed that doll therapy could act as a catalyst for initiating communication, offering a unique channel for expressive interaction for those struggling with conventional social exchanges. In another qualitative from the United States of America, a male veteran, Mr. B, experienced significant improvements in verbal communication and social interactions due to the presence of the doll [26]. Mr. B transitioned from making incomprehensible utterances to singing full songs while joyfully engaging with the doll, illustrating a marked enhancement in communication post intervention [26].

In addition to individual communication skills, this review suggests that doll therapy also positively impacts interactions with caregivers and family members. One South Korean study reported significant improvements in interactions between residents and staff, as well as with family members at care homes following doll therapy [30]. In other studies, it has been rereported that following doll therapy, residents frequently initiate conversations by asking questions like, ‘Who’s baby is this?’, which sparks dialogue and reminiscing about their own childhoods [21,23,24].

However, it is essential to acknowledge that not all interactions with the doll and caregivers result in positive outcomes. Two studies in this review report instances where residents refused to engage with the doll or expressed a dislike for it [29,30]. Despite these challenges, the studies also highlight the role of doll therapy in facilitating communication of instructions and tasks. For example, one study noted that staff in care homes use the doll as a visual aid to communicate instructions more effectively, leading to improved understanding and compliance among individuals with dementia [26].

It is also important to consider potential limitations in assessing the effects of doll therapy on communication. One study in this review raised concerns about the reliance on subjective impressions from nursing home staff rather than objective measures when assessing the impact of doll therapy [24]. The variability in caregiver assessments is due to the lack of standardized evaluation instruments in relation to doll therapy in dementia care.

Overall, this theme illustrates that doll therapy has significant potential to enhance communication among people living with dementia. By fostering meaningful connections and facilitating effective communication strategies, doll therapy appears to be a valuable therapeutic intervention in improving social engagement and promoting holistic wellbeing in care home settings.

#### 3.2.3. Theme 3: Reduction in Caregivers’ Distress

The third theme identified across the studies is the significant reduction in distress experienced by caregivers. The demanding nature of caregiving, particularly in managing behavioral disturbances among residents with dementia, often results in increased stress, negative emotions, and exhaustion. Several studies in this review suggested that doll therapy may be an effective intervention to alleviate caregiver burden associated with caring for people with dementia in care home settings [24,29,31].

These studies all report notable, statistically significant, decreases in caregiver distress, as measured by the Neuropsychiatric Inventory–Nursing Home (NPI-NH) Distress scale. This reduction in stress suggests that doll therapy not only benefits individuals with dementia but also enhances the overall quality of care by alleviating the strain on caregivers.

Additionally, one study links doll therapy to attachment theory, highlighting how it fosters feelings of security and nurturing caregiving relationships [31]. This approach may help mitigate attachment-related behaviors that can complicate interpersonal interactions. Doll therapy then appears to support the development of secure attachment bonds between residents with dementia and their caregivers, promoting a more supportive and nurturing environment within care homes [24,29,31]. This is further emphasized in randomized controlled trial carried out in Switzerland, whereby the authors highlighted the importance of comprehensive caregiver training to support therapeutic engagement with dolls in care homes. Proper training equips caregivers with the skills to manage behavioral challenges and foster secure attachment relationships, potentially enhancing the effectiveness and sustainability of doll therapy in dementia care settings.

Overall, the reduction in caregiver distress is a key finding, with doll therapy showing promise in easing the burden on caregivers. By fostering secure attachment bonds, improving caregiving relationships, and providing thorough training, doll therapy appears to be a valuable intervention for enhancing the wellbeing of both residents with dementia and their caregivers in care home settings.

## 4. Discussion

Non-pharmacological interventions are recommended as the primary treatment for behavioral and psychological symptoms of dementia (BPSD) according to longstanding guidelines from the National Institute for Health and Clinical Excellence—Social Care Institute for Excellence [4]. Despite this, the prevalent use of medications, particularly antipsychotics, among individuals with dementia in care homes remains a concern due to the higher likelihood of adverse outcomes such as sedation, polypharmacy, and increased risks of falls or cardiac arrest [33,34]. Clinical practice is gradually shifting towards prioritizing non-pharmacological interventions, acknowledging that pharmacological treatments should be secondary [35,36]. This change reflects a broader recognition of the need for safer, cost-effective, and efficacious approaches with minimal risks, as evidenced by the growing support for non-pharmacological strategies [37].

Among these non-pharmacological interventions, this review indicates that doll therapy appears to be a promising approach for people living with dementia in care home settings because it has the potential to reduce BPSD and improve overall wellbeing [22,23,24,25,26,27,28,29,30,31,32]. This therapy involves providing patients with dolls to create a caregiving relationship, promoting feelings of security and engagement [8]. Like other evidence-based interventions such as cognitive stimulation and creative therapeutic activities, the review findings support the notion that doll therapy can enhance the quality of life, mood, and social participation of amongst people living with dementia in care home settings [38]. The positive impact of doll therapy reported in the findings also aligns with Thomas Kitwood’s seminal person-centered dementia care theory, which emphasizes maintaining personhood in individuals with dementia and improving their wellbeing by enabling communication of their wants, needs, and desires, thus retaining their sense of identity [39,40,41]. This person-centered philosophy encourages care home staff to focus on building positive and enriching relationships to promote self-worth and reduce agitated behavior [42]. Beyond the philosophical benefits, doll therapy has also been associated with practical and clinical improvements in care homes, such as reductions in BPSD. This review highlights the therapy’s effectiveness in reducing challenging behaviors in people living with dementia. The link between doll therapy and the reduction in BPSD is supported by a growing evidence base, including guidelines and recommendations from authorities like NICE, which advocate for non-pharmacological interventions as initial strategies [34]. The clinical benefits of doll therapy are further substantiated by empirical research demonstrating its safe, cost-effective, and efficacious nature, with minimal risks and adverse effects [37].

Ethical considerations around doll therapy, once a contentious issue, appear to have significantly evolved. Earlier debates focused on the potential infantilization of individuals with dementia and concerns about treating them in a childish manner, which could reinforce stigmas associated with the condition [43,44]. These ethical dilemmas were prominent in previous reviews and critical commentaries [45,46]. However, this review reveals a notable shift in perspective, with fewer ethical limitations identified and a predominant focus on the positive outcomes of doll therapy. This change is likely due to the more robust empirical evidence supporting the therapy’s benefits, including numerous randomized controlled trials (RCTs) and studies with moderate to high-quality evidence presented in this review. While some studies highlighted the need for careful monitoring and appropriate training for caregivers to ensure the intervention’s safety and effectiveness, the overall consensus leans towards doll therapy’s therapeutic benefits outweighing potential risks. Ethical considerations remain relevant, but the focus has shifted towards maximizing benefits while ensuring the wellbeing and emotional comfort of individuals with dementia.

The evidence base for doll therapy has significantly progressed, with a notable increase in RCTs and studies of higher methodological quality compared to previous reviews [6,8,12]. The first systematic review on doll therapy by Mitchell et al. in 2014 [8] highlighted research studies primarily comprised qualitative studies without any RCTs identified, but this updated review, focusing on doll therapy in care home settings, identifies a substantial advancement in the empirical study of doll therapy. The inclusion of several robust RCTs and the use of validated evidence and tools have enhanced the methodological rigor of research in this area. Despite some inconsistencies in reporting, the adoption of standardized outcome measures and reporting guidelines improves the overall quality of research outcomes. The heterogeneity in reporting individual components of Neuropsychiatric Inventory (NPI) scores across studies remains a barrier to conducting a comprehensive meta-analysis. However, the potential for meta-analytical approaches to provide a robust assessment of doll therapy’s therapeutic effects on BPSD is significant. Future research should prioritize consistent reporting practices to enable meta-analyses, which could offer valuable insights into the intervention’s efficacy and inform clinical practice in dementia care homes. Additionally, future research should focus on developing and implementing best practice guidelines and education for using doll therapy in care home settings, with an emphasis on assessing its suitability for the individual needs of people living with dementia.

Overall, the findings of this review suggest a marked improvement in the empirical evidence supporting doll therapy, reflecting a shift from ethical concerns to a focus on maximizing therapeutic benefits. This development in evidence and perspective indicates a growing confidence in the efficacy of doll therapy as a non-pharmacological intervention in dementia care homes, providing a solid foundation for its continued use and development.

### 4.1. Implications for Nursing Practice

This scoping review on doll therapy interventions for people with dementia in care homes has several important implications for clinical nursing practice. The findings suggest that doll therapy can be an effective non-pharmacological intervention to manage behavioral and psychological symptoms of dementia (BPSD) [21,22,24,27,29,31,32]. For nurses, this indicates doll therapy could be a valuable tool to reduce agitation, aggression, and wandering behaviors without relying on medications. The review also highlighted increased communication skills and social interactions among residents receiving doll therapy [21,23,26,30]. This improved communication can aid nurses in better understanding residents’ needs and preferences, facilitating more person-centered care.

Additionally, this review found a reduction in caregiver distress associated with doll therapy interventions [24,29]. For nursing staff, this suggests that implementing doll therapy could lead to decreased work-related stress and burnout, potentially improving job satisfaction and quality of care. As a non-pharmacological approach aligned with person-centered care principles [21,25,26], doll therapy offers nurses an additional tool to manage BPSD while addressing individual needs and preferences. By incorporating doll therapy into their practice, nurses in care home settings can potentially improve outcomes for residents with dementia while also enhancing their own work experience and effectiveness. However, nurses should also be aware of ethical considerations and ensure that doll therapy is implemented appropriately and with respect for each resident’s dignity and autonomy.

### 4.2. Strengths and Limitations

Strengths of this review include a comprehensive search strategy that utilized four major electronic databases (CINAHL, Medline, PsycINFO, and PubMed) to ensure wide coverage of the relevant literature. This review adhered to rigorous methodology, following established guidelines for scoping reviews, including those from the Joanna Briggs Institute, Arksey and O’Malley, and PRISMA-ScR [14,15,16]. By focusing on studies published in the last ten years, this review ensured the examination of the most current evidence. It included various types of empirical studies and evidence reviews, allowing for a comprehensive overview of the topic. The narrative synthesis approach integrated both qualitative and quantitative data, providing a flexible method for summarizing and interpreting research findings. However, this review has limitations, such as the inclusion of only studies written in English, potentially missing relevant research in other languages. Despite no geographical restrictions, the language limitation might have resulted in a bias towards English-speaking countries. The focus on care homes excluded studies conducted in acute care or hospital-based settings, potentially limiting the overall perspective on doll therapy. There is also a risk of publication bias, where studies with positive results are more likely to be published and included. Additionally, the heterogeneity of the included studies may have made direct comparisons challenging.

## 5. Conclusions

This scoping review provides valuable insights into the impact of doll therapy for individuals with dementia in care home settings. The evidence suggests that doll therapy can be an effective non-pharmacological intervention for reducing behavioral and psychological symptoms of dementia (BPSD), improving communication skills, and potentially reducing caregiver distress. This review highlights the potential of doll therapy to enhance the quality of life for individuals living with dementia by promoting feelings of security, engagement, and improved social interactions. In conclusion, doll therapy appears to be a valuable tool in the arsenal of non-pharmacological interventions for dementia care in care homes. Its potential to reduce BPSD and improve quality of life warrants further exploration and consideration in care planning for individuals with dementia. As the global prevalence of dementia continues to rise, such person-centered, non-invasive approaches will become increasingly important in providing compassionate and effective care.

## Figures and Tables

**Table 1:** Characteristics of studies included in this review.

Authors	Country	Aim of Research	Research Design	Sample and Setting	Main Findings
Alander et al., 2018	England	Explore how people in care, doll users and non-doll users, make sense of doll use in their settings.	Grounded theory	3 care homes, 16 participants	Four main themes emerged: intrapersonal features, interpersonal features, behavioral benefits, and ethical and moderating factors. Residents generally support the use of dolls, believing that dolls can have a positive impact.
Balzotti et al., 2019	Italy	Compare the effects in older individuals with dementia, of two intervention programs, gesture verbal treatment and doll therapy.	RCT	1 care home, 35 participants	In assessing twelve neuropsychiatric symptom subtypes, significant changes were revealed in three. Verbal therapy demonstrated superior efficacy in reducing apathy, while doll therapy exhibited greater effectiveness in reducing agitated behaviors and irritability, persisting even when accounting for baseline agitation scores.
Braden et al., 2018	USA	Evaluate the implementation of doll therapy on the occurrence of agitated behaviors of individuals with dementia.	Non-RCT	1 care home, 16 participants	Participants had a higher level of happiness, activity, interaction with staff and others, and ease of caregiving.
Cantarella et al., 2018	Italy	Measure the impact of doll therapy on people with severe dementia.	RCT	1 care home, 29 participants	The doll therapy (DT) group exhibited lower post-test scores, indicating a reduction in behavioral and psychological symptoms of dementia (BPSD) compared to the control group. Additionally, the DT group showed lower Neuropsychiatric Inventory (NPI)-related caregiver distress scores.
Cohen-Mansfield et al., 2015	Israel	Comparison of different nonpharmacological interventions for persons with BPSD.	Exploratory	6 nursing homes, 89 participants	Doll therapy was amongst the most highly utilized interventions with 369 sessions and 64 people.
Malinowski et al., 2022	USA	To observe the emotional, behavioral, and social response to doll therapy in male veterans.	Observational study	1 day center 2 participants	An increase in emotional response, behavioral response, and social response in people with dementia. Findings indicate the effects of dolls in male veterans similar to those found in female civilians.
Molteni et al., 2022	Switzerland	To examine the efficacy of doll therapy in reducing BPSD, professional caregiver distress, and patients’ biomarkers of stress.	RCT	26 nursing homes, 129 participants	Doll therapy intervention (DTI) group exhibited a greater reduction in agitation compared to the standard intervention (SI) group, along with a significant decrease in Neuropsychiatric Inventory—Nursing Home (NPI-NH) distress net change.
Moyle et al., 2018	Australia	To compare a lifelike baby doll intervention for reducing anxiety, agitation, and aggression in older people with dementia.	Mixed methods	5 long-term facilities	No significant group or time effects on various outcomes, except for a significant group-by-time interaction effect for pleasure. The lifelike doll group showed significantly more pleasure than the usual care group at week 3 compared to baseline, with a clinically meaningful effect size.
Santagata et al., 2021	Italy	Evaluating the effect of doll therapy in the management of BPSD, reduction in caregiver burden and delirium incidences.	RCT	2 nursing homes, 52 participants	Focused on primary outcomes, aiming to reduce BPSD and professional caregiver burden, with a secondary goal of decreasing delirium incidence. While the DT group did not significantly differ from the standard treatment (ST) group in various parameters, professional caregiver burden was significantly higher in the DT group. Notably, DT effectively calmed patients in 87.5% of instances, with a significant decrease in delirium incidence.
Shin et al., 2015	Korea	To examine the effects of dolls on the physical, emotional and psychological facets of individuals with dementia.	Pre and Post test	1 nursing home, 51 participants	Statistically significant reduction in aggression, wandering. Statistically significant improvement in verbalization, positive mood, and decrease in depression.
Vaccaro et al., 2020	Switzerland	To identify the efficacy of doll therapy versus a sham intervention.	RCT	22 nursing homes, 128 participants	Primary outcomes—decrease in patients’ BPSD, decrease in the professional caregiver’s distress, interactions with the doll.
Yilmaz et al., 2021	Turkey	To identify the effect of doll therapy on agitation and cognitive state in institutionalized patients with moderate to severe dementia.	RCT	1 nursing home, 29 participants	Statistically significant improvement in agitation levels and other behavioral disturbances in the intervention group.

## Data Availability

The datasets generated and/or analyzed during the current study are available from the corresponding author upon reasonable request.

## Supplementary Materials

The following supporting information can be downloaded at: https://www.mdpi.com/article/10.3390/nursrep14040200/s1, Table S1: Preferred reporting items for systematic reviews and Meta-Analyses extension for scoping reviews (PRISMA-ScR) checklist; Table S2: Example of search results from clinical.

## Public Involvement Statement

No public involvement in any aspect of this review.

## Guidelines and Standards Statement

This manuscript was drafted against the PRISMA extension for Scoping Reviews (https://www.equator-network.org/reporting-guidelines/prisma-scr/ (Accessed on 21 September 2024)).
