# Supplementary material for: Therapeutic Doll Interventions for People Living with Dementia in Care Homes: A Scoping Review
Source: Nurs Rep. 2024 Oct 1;14(4):2706–18. doi: 10.3390/nursrep14040200 (PMC11503298; doi:10.3390/nursrep14040200)
Supplement: Supplementary file 1 [file nursrep-14-00200-s001.zip › Supplementary Material S2 .pdf]

Supplementary Material S2: Example of Search Results from CINAHL

Search ID	Search Terms	Number of Results
S14	S6 AND S13	63
S13	S7 OR S8 OR S9 OR S10 OR S11 OR S12	555
S12	"empathy doll"	8
S11	"baby doll"	13
S10	"soft toy"	8
S9	"dolls"	217
S8	"doll"	367
S7	"doll therapy"	53
S6	S1 OR S2 OR S3 OR S4 OR S5	117,075
S5	"cognitive impairment"	45,394
S4	"living with dementia"	2,641
S3	"people with dementia"	8,051
S2	(MH "Alzheimer's Disease")	38,610
S1	(MH "Dementia")	47,763
